# Serum glycobiomarkers for chronic inflammatory demyelinating polyneuropathy

**DOI:** 10.1111/ene.70023

**Published:** 2024-12-25

**Authors:** Soma Furukawa, Yuki Fukami, Hisatoshi Hanamatsu, Ikuko Yokota, Jun‐ichi Furukawa, Masaya Hane, Ken Kitajima, Chihiro Sato, Keita Hiraga, Yuki Satake, Satoru Yagi, Haruki Koike, Masahisa Katsuno

**Affiliations:** ^1^ Department of Neurology Nagoya University Graduate School of Medicine Nagoya Aichi Japan; ^2^ Institute for Glyco‐core Research (iGCORE) Nagoya University Nagoya Aichi Japan; ^3^ Department of Orthopaedic Surgery, Faculty of Medicine and Graduate School of Medicine Hokkaido University Sapporo Hokkaido Japan; ^4^ Division of Neurology, Department of Internal Medicine, Faculty of Medicine Saga University Saga Japan; ^5^ Department of Clinical Research Education Nagoya University Graduate School of Medicine Nagoya Aichi Japan

**Keywords:** chronic inflammatory demyelinating polyneuropathy, glycans, glycosylation, intravenous immunoglobulin

## Abstract

**Background:**

This study conducted a comprehensive glycan analysis of serum to determine how glycan biomarkers are associated with the pathophysiology of chronic inflammatory demyelinating polyneuropathy (CIDP) and the effects of its treatment.

**Methods:**

We comparatively analyzed *N*‐ and *O*‐glycans in the pretreatment serum of 27 treatment‐naïve patients with typical CIDP and 20 age‐ and sex‐matched healthy controls (HC) using mass spectrometry. We determined the association between clinical parameters and glycans. The serum glycan and neurofilament light‐chain (NfL) levels were assessed at the baseline, and treatment response was defined according to the degree of improvement in the modified Rankin scale 12 weeks after the first dose of intravenous immunoglobulin (IVIg).

**Results:**

Compared with the HC, the CIDP group demonstrated significantly lower levels of serum total *N*‐glycans (CIDP, median 973.3 [IQR 836.2–1131.3] pmol/μL; HC, 1125.0 [1005.0–1236.2] pmol/μL; *p* < 0.05), especially sialylated *N*‐glycans (CIDP, 898.0 [752.2–1037.2] pmol/μL; HC, 1064.4 [942.7–1189.8] pmol/μL; *p* < 0.01). In contrast, the *O*‐glycan levels did not differ significantly between the two groups. The treatment response was associated with low *N*‐glycan levels, but not with the serum NfL levels. Low levels of sialylated *N*‐glycans were associated with resistance to treatment over 12 weeks, with an area under the curve of 0.822 (*p* < 0.01).

**Conclusions:**

Low levels of sialylated *N*‐glycans could potentially serve as a novel biomarker, reflecting pathophysiology and therapeutic resistance in typical CIDP.

## INTRODUCTION

Chronic inflammatory demyelinating polyneuropathy (CIDP) is an acquired immune‐mediated disorder with a pathogenesis that remains poorly understood [1]. Intravenous immunoglobulin (IVIg) therapy is a first‐line treatment for induction and maintenance phases [2]. However, a subset of patients exhibits suboptimal responses to IVIg, leading to a chronic progressive course resulting in substantial neurological impairment and diminished quality of life [3].

Neurofilament light chains (NfL) have emerged as potential biomarkers associated with disease activity in CIDP, but no biomarker has yet been validated to reliably reflect the underlying pathophysiology or predict treatment responses in clinical practice [4, 5, 6, 7, 8]. This highlights the critical need for identifying biomarkers that can provide insights into disease mechanisms and guide therapeutic strategies.

Glycan modifications, particularly in the IgG‐Fc region, have been implicated in the pathogenesis of CIDP [9]. Prior studies have suggested that changes in IgG‐Fc glycans, particularly a proportional decrease in sialylated glycans, correlate with disease severity and response to therapy [10]. Sialic acid‐containing glycoproteins, abundant in peripheral and central nervous systems, play a key role in modulating immune responses through mechanisms involving complement inhibition and microglial activity regulation via complement regulatory factors [11, 12, 13].

In murine models of demyelination, a reduction in *N*‐glycans has been shown to exacerbate inflammatory demyelination [14]. Moreover, recent evidence indicates a decrease in *N*‐acetylglucosamine (GlcNAc), a precursor of *N*‐glycan branching, in the sera of patients with progressive multiple sclerosis, with levels correlating with disease severity [15, 16]. These findings suggest that similar glycan alterations may also be relevant in CIDP. However, the changes in the levels of serum glycans in CIDP have not yet been verified.

In this study, we aimed to conduct a comprehensive glycan analysis of serum samples from CIDP patients to identify potential glycan biomarkers that are associated with therapeutic efficacy.

## METHODS

### Standard protocol approvals, registrations, and patient consents

This study was performed in accordance with the Declaration of Helsinki and the Ethical Guidelines for Medical and Health Research Involving Human Subjects endorsed by the Japanese government, with approval from the Ethics Review Committee of Nagoya University Graduate School of Medicine (No. 2014‐0424 and 2019‐0170). Before participating in the research, written informed consent from all participants was obtained after receiving the content information endorsed by the Ethics Review Committee, including the unfavorable and favorable aspects of the study and other relevant data.

### Participants

This study included consecutive patients with confirmed diagnoses of typical CIDP, as defined by the European Academy of Neurology/Peripheral Nerve Society guidelines 2021 criteria, at Nagoya University Hospital between July 2012 and August 2021 [17]. Most patients were directed from other hospitals for further diagnostics and therapy, and CIDP was diagnosed by two neurologists (S.F. and Y.F.). We measured the antibodies against paranodal cell adhesion molecules (i.e., contactin‐1 [CNTN1], neurofascin‐155 [NF155], and contactin‐associated protein1 [Caspr1]) in all patient sera using enzyme‐linked immunosorbent assays in our laboratory [18]. Patients with CIDP variants or autoimmune nodopathies expressing positive anti‐CNTN1, NF155, or Caspr1 antibodies were excluded from the study. Furthermore, patients who had been subjected to therapy with immunomodulatory agents (i.e., IVIg, plasma exchange, corticosteroids, or immunosuppressive drugs) before baseline were excluded. The age‐ and sex‐matched HC comprised the comparison group. The exclusion criteria for the control group included brain injuries and neurological diseases in medical history.

### Sample collection and serum NfL analysis

Serum was collected from patients on admission or attendance at the Nagoya University Hospital. The blood tubes were promptly removed to the laboratory department of the hospital and subjected to centrifugation within 1 h following collection. The serum was frozen and preserved at −80°C until use. The serum NfL protein concentrations were measured in duplicate using the Simoa HD‐1 Analyzer (Quanterix, Lexington, MA, USA) and ultrasensitive paramagnetic bead‐based enzyme‐linked immunosorbent assay as performed by researchers blinded to all clinical data.

### Extraction, purification, and analysis of serum *N*‐ and *O*‐glycans

The releasing of *N*‐glycan from serum proteins was performed according to previous reports [19]. Released *N*‐glycans were purified by glycoblotting procedures combining with sialic acid linkage‐specific alkylamidation (SALSA) [20, 21]. After removal of excess reagents, *N*‐glycans labeled with aoWR were subjected to matrix‐assisted laser desorption‐ionization time of flight mass spectrometry (MALDI‐TOF MS) analysis. In *O*‐glycomic analysis, the sialic acid linkage‐specific derivatization was performed according to the previous report [21]. Briefly, secretor proteins in serum (10 μL) were derivatized in a linkage‐specific manner using the SialoCapper‐ID Kit. Excess SALSA reagents were removed from SALSA‐derivatized serum proteins by acetonitrile precipitation. *O*‐Glycans were prepared by β‐elimination in the presence of pyrazolone (BEP) method with minor modifications [22]. Derivatized *O*‐glycans were subjected to MALDI‐TOF MS analysis.

### Clinical evaluations

We evaluated the muscle strength using the Medical Research Council (MRC) sum score and disease severity using the modified Rankin Scale (mRS). The MRC sum score was used to assess the strength of six bilateral (left and right) muscle groups on a scale from 0 (no visible contraction) to 5 (normal muscle strength), resulting in a total score from 0 to 60. Treatment response was assessed based on improvement at 2, 4, and 12 weeks after the initial administration of IVIg (400 mg/kg/day for 5 days) compared to baseline. The group with no improvement in the mRS score was categorized as the non‐responder group, while the group with an improvement of 1 or more was categorized as the responder group. Although no patient underwent additional therapy within the first 2 weeks after the initial treatment, 15 out of 27 patients received additional treatment beyond 4 weeks, even in the absence of clinical deterioration. Therefore, treatment responses were also assessed at 2 and 4 weeks post‐treatment.

The patients underwent nerve conduction studies on four motor nerves (median, ulnar, tibial, and peroneal nerves) and three sensory nerves (median, ulnar, and sural nerves) with surface stimulation and recording electrodes according to standard protocols. The parameters analyzed for the motor nerves included distal latency, compound muscle action potential (CMAP) amplitude, and conduction velocity, whereas those for the sensory nerves covered sensory nerve action potential (SNAP) amplitude and conduction velocity. The CMAP and SNAP amplitudes were evaluated from baseline to the first negative peak.

### Statistical analysis

The *m*/*z* values were used to generate the compositions of glycans detected by tandem MALDI‐TOF MS and create spectra. The descriptive statistics was expressed as median (interquartile range [IQR]) for nonparametric continuous data. The intergroup comparisons of the latter values were performed using Mann–Whitney *U*‐test. The differences in categorical variables were analyzed with chi‐squared test. The receiver operating characteristic (ROC) curves were evaluated for the patients with CIDP and healthy controls (HC) along with the non‐responder and responder groups, and the area under the curve (AUC) values were computed. Multiple comparisons were conducted using the analysis of variance with Kruskal–Wallis test, and if a significant difference was observed, pairwise tests were performed between the two groups, with the significant probabilities corrected by Bonferroni adjustment. The Spearman rank correlation coefficient was used to estimate the correlation between parameters. *p*‐values of <0.05 and correlation coefficient (*r*) >0.3 were regarded as statistically significant. Statistical calculations were performed using the Statistical Package for the Social Sciences V.29.0J software (IBM Japan, Tokyo, Japan).

## RESULTS

### Baseline characteristics of the participants

Of the 81 consecutive patients with CIDP, we excluded 9 with autoimmune nodopathies, 33 who had been administered with immunomodulatory agents prior to baseline, and 12 with CIDP variants. Eventually, 27 treatment‐naïve cases of typical CIDP and 20 age‐ and sex‐matched HC were included (Figure S1).

Table 1 shows the clinical backgrounds of patients with CIDP and HC. The median mRS for patients with CIDP at baseline was 3. The patients with CIDP had significantly higher serum NfL levels than the HC (*p* < 0.05). All patients (27 cases, 100%) received IVIg as the initial therapy. Over the 12‐week period, IVIg was administered once in 12 cases (44%), twice in 11 cases (41%), and three or more times in 4 cases (15%). In addition to IVIg, some patients received adjunctive therapies: intravenous methylprednisolone was administered in 2 cases (7%), daily oral prednisolone in 3 cases (11%), and plasma exchange in 1 case (4%).

**TABLE 1:** Characteristics of patients with CIDP and healthy controls.

	Typical CIDP *n* = 27	Control *n* = 20	*p*‐value
Age at sampling, years (median [IQR])	56 (36–69)	66 (29–72)	NS
Sex, female	13 (48%)	9 (45%)	NS
Diabetes	1 (4%)	0 (0%)	NS
Disease duration, m (median [IQR])	6 (4–14)	NA	
MRC sum score	44 (38‐53)	ND	
mRS	3 (2–4)	0 (0–0)	<0.01
Serum NfL levels, pg/mL (median [IQR])	13.2 (10.1–41.7)	12.4 (5.9–17.3)	<0.05
CSF findings			
Cell, /μlL(median [IQR])	1 (1–2)	ND	
Protein, mg/dL (median [IQR])	73 (49–101)	ND	
Motor conduction studies (median [IQR])			
Median nerve			
DL, ms	5.0 (3.9–6.1)	ND	
CMAP amplitude, mV	6.2 (3.7–7.7)	ND	
MCV, m/s	35.0 (29.0–45.0)	ND	
Ulnar nerve			
DL, ms	3.4 (3.0–4.2)	ND	
CMAP amplitude, mV	5.7 (4.3–7.3)	ND	
MCV, m/s	41.0 (32.0–53.0)	ND	
Tibial nerve			
DL, ms	5.3 (4.7–6.2)	ND	
CMAP amplitude, mV	5.2 (2.9–7.9)	ND	
MCV, m/s	37.0 (31.5–42.5)	ND	
Peroneal nerve			
DL, ms	5.7 (4.9–6.8)	ND	
CMAP amplitude, mV	1.7 (0.4–2.3)	ND	
MCV, m/s	36.0 (32.3–43.0)	ND	
Sensory conduction studies (median [IQR])			
Median nerve			
SNAP amplitude, μV	7.1 (1.8–11.4)	ND	
SCV, m/s	44.4 (40.5–47.0)	ND	
Ulnar nerve			
SNAP amplitude, μV	8.3 (0.4–14.4)	ND	
SCV, m/s	41.0 (36.5–44.0)	ND	
Sural nerve			
SNAP amplitude, μV	13.2 (2.9–21.8)	ND	
SCV, m/s	44.1 (42.0–48.9)	ND	

Abbreviations: CIDP, chronic inflammatory demyelinating polyradiculoneuropathy; CMAP, compound muscle action potential; CSF, cerebrospinal fluid; DL, distal latency; IQR, interquartile range; IVIg, intravenous immunoglobulin; MCV, motor nerve conduction velocity; MRC, Medical Research Council Scale; mRS, modified Rankin scale; NA, not applicable; ND, not detected; NfL, neurofilament light chain; NS, not significant; SCV, sensory nerve conduction velocity; SNAP, sensory nerve action potential.

The degree of improvement in mRS compared with baseline values was evaluated at 2, 4, and 12 weeks after the initial therapy. At 2 weeks, improvement was 0 in 9 cases (34%), 1 in 12 cases (44%), and 2 in 6 cases (22%). At 4 weeks, improvement was 0 in 8 cases (30%), 1 in 12 cases (44%), and 2 in 7 cases (26%). At 12 weeks, improvement was 0 in 8 cases (30%), 1 in 12 cases (44%), 2 in 6 cases (22%), and 3 in 1 case (4%). No patient showed an improvement of ≥4 over the 12‐week period.

### Profiles of serum *N*‐ and *O*‐type glycans by mass spectrometry analysis

**FIGURE 1:**
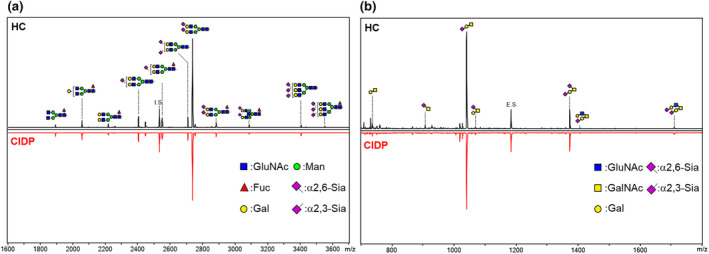
Representative serum *N*‐ and *O*‐glycan profiles of patients with CIDP and controls. Representative matrix‐assisted laser desorption‐ionization time of flight mass spectrometry spectra of *N*‐glycans (a) and *O*‐glycans (b) in healthy controls (HC) and patients with CIDP sera. Each peak was assigned a representative glycan structure.

In patients with CIDP and HC, 59 serum *N*‐type and 18 serum *O*‐type glycans were found (Figure 1a,b). The former were classified into 5‐high mannose, 39‐sialylated, and 15‐neutral types, whereas the latter were categorized into 11‐sialylated and 7‐neutral types. The sialylated glycans were divided into α2,3‐, α2,6‐, and α2,3−/α2,6‐linked types according to their binding patterns. The most frequently observed *N*‐glycan was the α2,6‐linked sialylated type, Hex_2_HexNAc_2_NeuAc_2_ [α2,6/α2,6] + Man_3_GlcNAc_2_, and the most abundant *O*‐glycan was α2,3‐linked type, HexNAc_1_Hex_1_NeuAc_1_ [α2,3] (Tables S1 and S2).

### Comparison of the serum *N*‐ and *O*‐glycan levels between the HC and patients with CIDP

**FIGURE 2:**
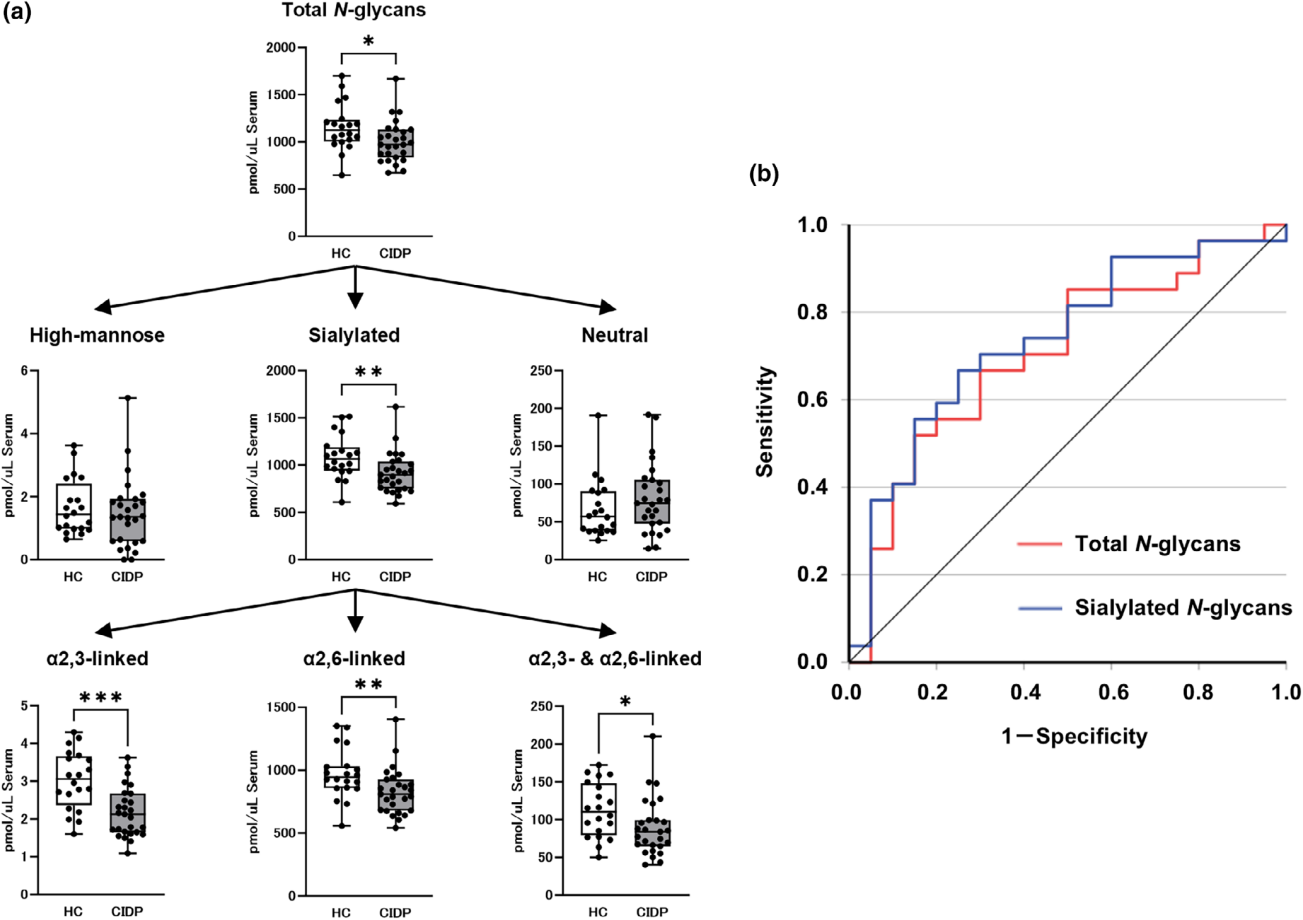
Serum *N*‐glycans levels in patients with CIDP and controls. (a) Serum *N*‐glycans levels in the HC and patients with CIDP. (b) Receiver operating characteristic (ROC) analysis discriminating patients with CIDP from HC showed an area under the curve (AUC) for serum total *N*‐glycans and sialylated *N*‐glycans levels being 0.704 and 0.735, respectively. Horizontal lines in the boxplots indicate the median. The top and bottom edges of each box indicate the IQR. The I‐bar indicates the range between the minimum and maximum values. Statistical analysis was performed using the Mann–Whitney *U*‐test. *p < 0.05, **p < 0.01, ***p < 0.001.

The serum total *N*‐glycans levels of patients with CIDP were significantly lower than in the HC (CIDP, median 973.3 [IQR 836.2–1131.3] pmol/μL; HC, 1125.0 [1005.0–1236.2] pmol/μL; *p* < 0.05; Figure 2a). In the subgroup analysis, the sialylated *N*‐glycans levels were significantly lower in the patients with CIDP than in the HC (CIDP, 898.0 [752.2–1037.2] pmol/μL; HC, 1064.4 [942.7–1189.8] pmol/μL; *p* < 0.01), although the high mannose or neutral types did not differ significantly (Figure 2a). The levels of α2,3‐, α2,6‐, and α2,3−/α2,6‐linked sialylated *N*‐glycans were all significantly lower in the patients with CIDP than in the HC (Figure 2a). The ROC curve indicated that the serum total *N*‐glycans, specifically sialylated types, distinguish the patients with CIDP from the HC, with the respective AUC values of 0.704 (95% confidence interval [CI], 0.551–0.856, *p* < 0.05) and 0.735 (95% CI, 0.589–0.881, *p* < 0.01) (Figure 2b).

**FIGURE 3:**
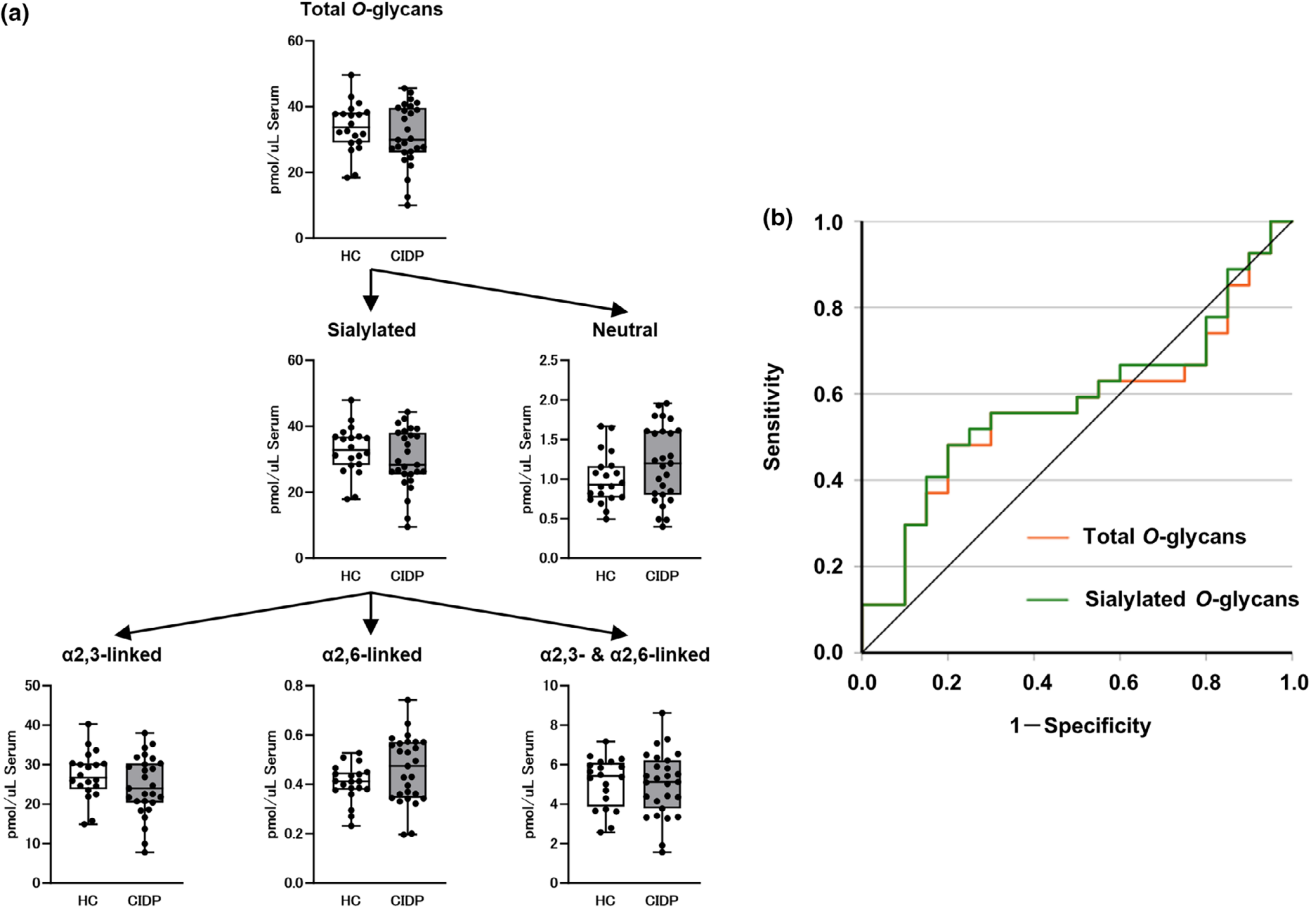
Serum *O*‐glycans levels in patients with CIDP and controls. (a) Serum *O*‐glycans levels in the HC and patients with CIDP. (b) ROC analysis revealed that the serum total *O*‐glycans levels did not contribute to the discrimination between patients with CIDP and HC. The top and bottom edges of each box indicate the IQR. The I‐bar indicates the range between the minimum and maximum values. Statistical analysis was performed using the Mann–Whitney *U*‐test. No statistically significant difference was shown in any comparison.

The serum total *O*‐glycans levels did not differ significantly between the CIDP and HC patients (Figure 3a). The subgroups of *O*‐glycans, including the sialylated glycans, showed a similar trend (Figure 3a). Furthermore, the ROC curves did not contribute to the discrimination between the patients with CIDP and HC with AUC values of 0.569 (95% CI, 0.402–0.735, *p* = 0.426) for total *O*‐glycans and 0.581 (95% CI, 0.416–0.747, *p* = 0.344) for sialylated *O*‐glycans (Figure 3b).

### Association between the serum *N*‐ and *O*‐glycan levels and treatment response

**FIGURE 4:**
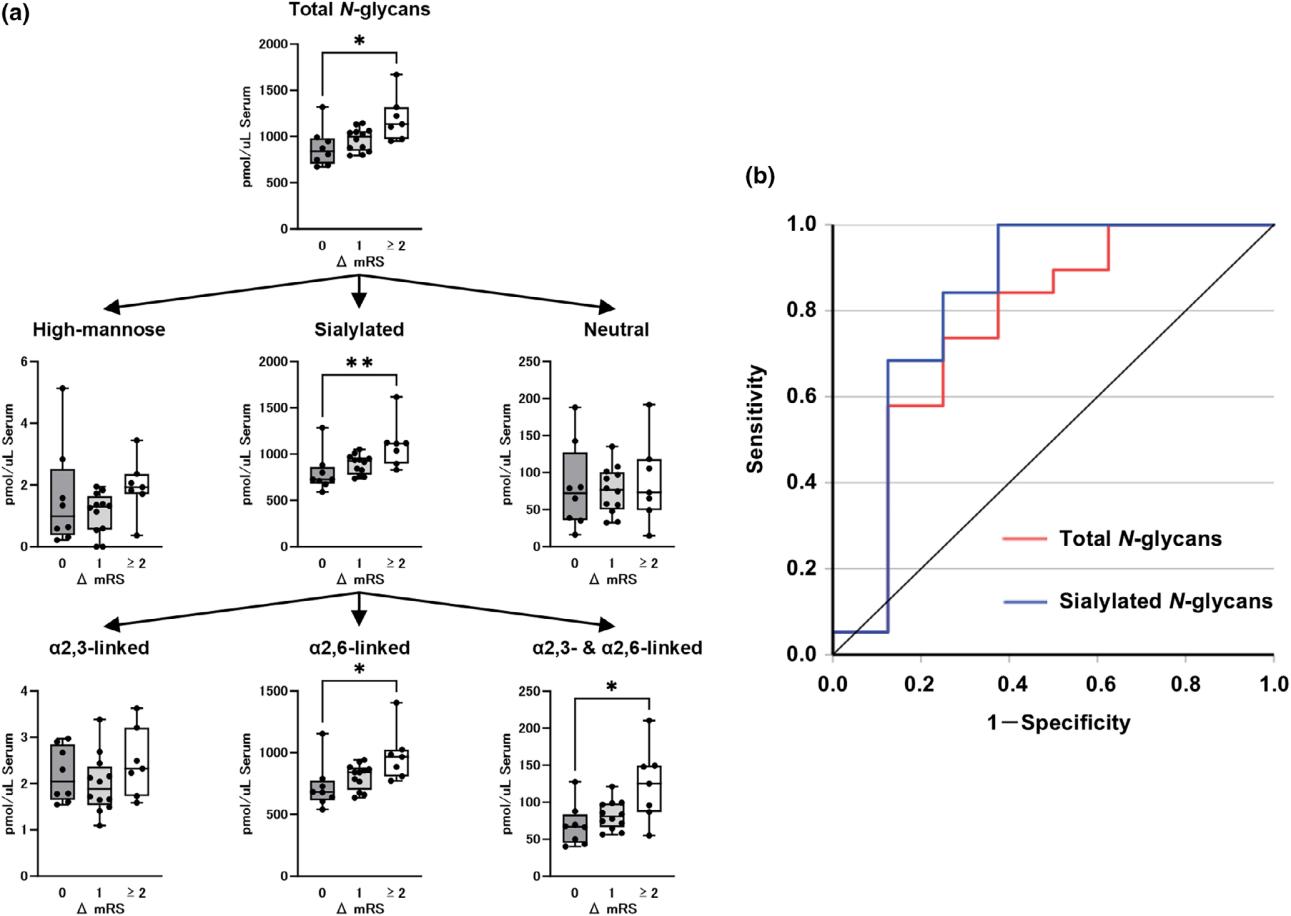
Association of serum *N*‐glycans levels with response to treatment. (a) Association between the serum *N*‐glycans levels and mRS improvement score from baseline after 12 weeks of initial treatment in patients with CIDP. (b) ROC analysis differentiating the responder group from the non‐responder group showed that the AUC for the serum total *N*‐glycans and sialylated *N*‐glycans levels were 0.763 and 0.822, respectively. The top and bottom edges of each box indicate the IQR. The I‐bar indicates the range between the minimum and maximum values. *p < 0.05, **p < 0.01, multiple comparisons using Bonferroni correction. ΔmRS, degree of mRS improvement after 12 weeks of initial treatment.

To determine whether the serum glycan levels are associated with responses to treatment, we analyzed the correlation between baseline glycan levels and improvement in mRS scores during the 12‐week period after initial therapy. A significant difference in serum total *N*‐glycans levels was observed among the three groups with mRS improvement scores of 0, 1, and ≥2 (840.4 [705.3–980.9], 997.9 [846.3–1058.9], and 1133.5 [973.3–1317.5], respectively, *p* < 0.05) (Figure 4a). After adjusting for multiple comparisons using Bonferroni correction, a significant difference was observed between Groups 0 and 2 (*p* < 0.05), indicating that lower *N*‐glycan levels were associated with therapeutic resistance over 12 weeks. In the subgroup analysis, the significant difference in the sialylated glycans levels was observed among the three groups (Figure 4a). There were no significant differences among groups in the high mannose and neutral types (Figure 4a). In the sialylated glycans, the α2,6‐ and α2,3−/α2,6‐linked types, but not the α2,3‐linked types, demonstrated a significant difference (Figure 4a). The ROC curve indicated that the serum total *N*‐glycans, specifically the sialylated types, were distinguishable between the non‐responder and responder groups, with respective AUC values of 0.763 (95% CI, 0.536–0.990, *p* < 0.05) and 0.822 (95% CI, 0.600–1.000, *p* < 0.01) (Figure 4b). Analysis of the association between serum *N*‐glycan levels and improvement in mRS scores at 2 and 4 weeks after initial therapy showed similar results, though no significant difference in α2,3−/α2,6‐linked sialylation was observed between the groups at either time point (Figures S2 and S4).

**FIGURE 5:**
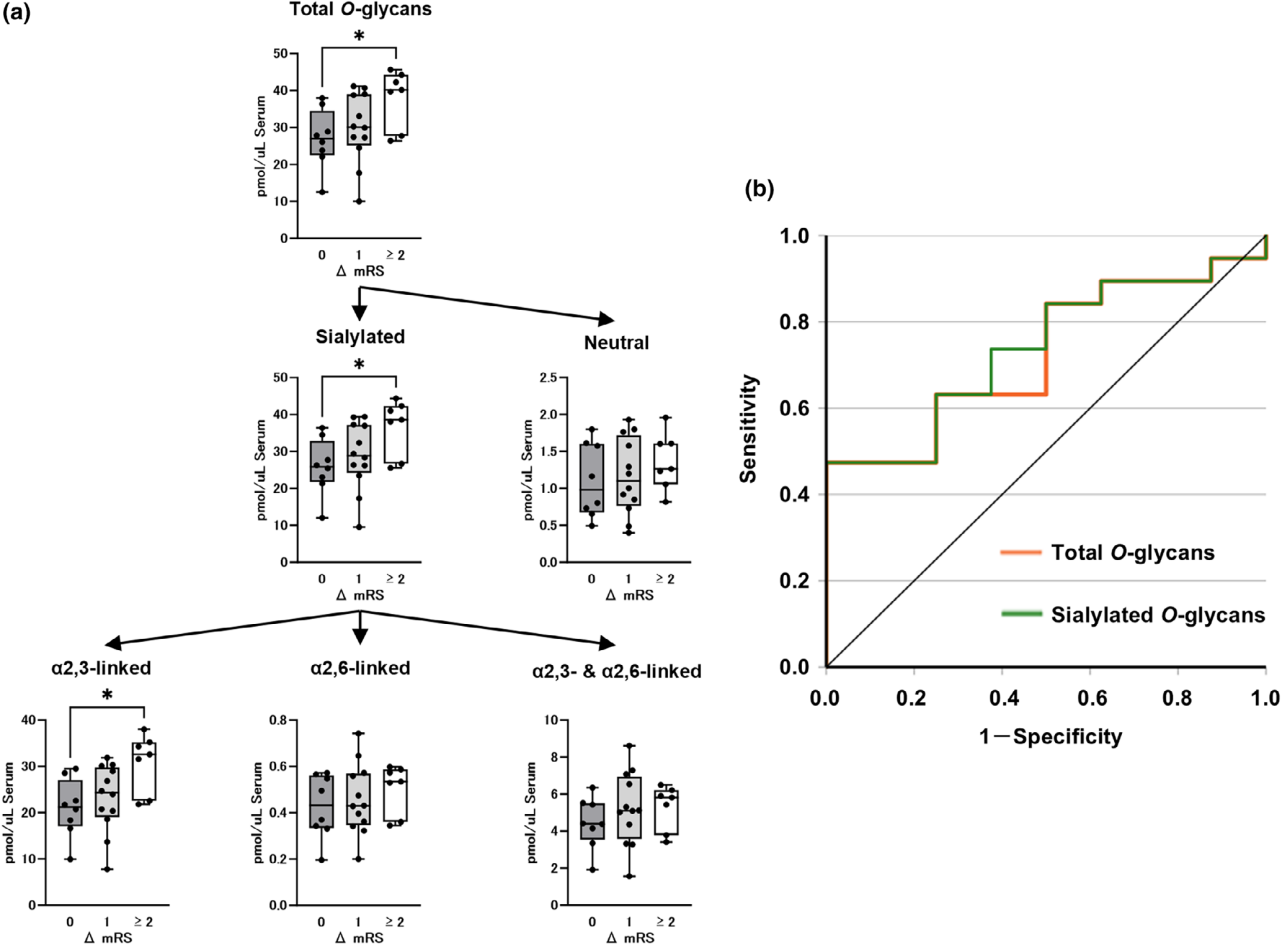
Association of serum *O*‐glycans levels with response to treatment. (a) Association between the serum *O*‐glycans levels and mRS improvement score from baseline after 12 weeks of initial treatment in patients with CIDP. (b) ROC analysis to discriminate the responder group from the non‐responder group showed that the AUC for the serum total *O*‐glycans and sialylated *O*‐glycans levels were 0.724 and 0.737, respectively. The top and bottom edges of each box indicate the IQR. The I‐bar indicates the range between the minimum and maximum values. *p < 0.05, multiple comparisons using Bonferroni correction. ΔmRS, degree of mRS improvement after 12 weeks of initial treatment.

There was a significant difference in serum total *O*‐glycans levels among the three groups with mRS improvement scores of 0, 1, and ≥2 (27.0 [22.5–34.5], 30.1 [25.2–39.0], and 40.2 [27.7–44.3], respectively, *p* < 0.05) (Figure 5a). Multiple comparisons revealed a significant difference between Groups 0 and 2 (*p* < 0.05). This indicated that lower *O*‐glycan values, similar to *N*‐glycans, were suggestive of therapeutic resistance. In the subgroup analysis, sialylated but not neutral glycans differed significantly among the three groups (Figure 5a). A significant difference was observed in the α2,3‐linked type, but not in the α2,6‐ and α2,3−/α2,6‐linked types (Figure 5a). Although without statistical significance, the ROC curve showed that the serum total *O*‐glycans and sialylated *O*‐glycans discriminated the non‐responder and responder groups, with AUC values of 0.724 (95% CI, 0.529–0.918, *p* = 0.071) and 0.737 (95% CI, 0.546–0.927, *p* = 0.056), respectively (Figure 5b). Analysis of the association between serum *O*‐glycan levels and improvement in mRS scores at 2 and 4 weeks after initial therapy showed similar results, except that the ROC curve at both time points indicated that the serum total *O*‐glycans and sialylated *O*‐glycans could accurately discriminate between the non‐responder and responder groups (Figures S3 and S5).

We analyzed the impact of other factors on the treatment responsiveness (Figure S4). Regarding the factors such as age and duration of illness, no significant differences were found among the three groups categorized by mRS improvement scores of 0, 1, and 2. Similarly, no significant differences in the levels of cerebrospinal fluid protein and CMAP amplitudes of the median, ulnar, peroneal, and tibial nerves were observed among the groups. Moreover, no significant difference in the serum NfL levels was observed among the groups.

### Correlation between the serum *N*‐ and *O*‐glycans and electrophysiological indices

In the electrophysiological findings, a correlation was observed between the serum total *N*‐glycans levels and distal latency of peroneal nerve (Table 2). Furthermore, a correlation was observed between the serum total *O*‐glycans levels and SCV of the sural nerve. Correlations were also observed between the serum NfL levels and several indices of nerve conduction: CMAP amplitude of the median, ulnar, or peroneal nerves; distal latency of the tibial nerve; MCV of the tibial and peroneal nerves; and SNAP amplitude and SCV of the sural nerve.

**TABLE 2:** Correlation of nerve conduction studies with serum *N*‐ or *O*‐glycan and neurofilament levels.

		*N*‐glycans		*O*‐glycans		NfL	
		*rs*	*p*	*rs*	*p*	*rs*	*p*
DL (ms)	Median nerve	−0.315	0.110	−0.167	0.405	0.164	0.413
	Ulnar nerve	−0.190	0.341	0.017	0.935	0.271	0.172
	Tibial nerve	−0.158	0.450	0.013	0.949	−0.501	0.011
	Peroneal nerve	−0.524	0.010	−0.160	0.466	−0.411	0.052
CMAP amp. (mV)	Median nerve	0.087	0.665	0.139	0.489	−0.455	0.017
	Ulnar nerve	0.122	0.544	0.067	0.739	−0.501	0.008
	Tibial nerve	0.109	0.587	0.000	0.999	−0.361	0.064
	Peroneal nerve	0.162	0.421	0.212	0.288	−0.507	0.007
MCV (m/s)	Median nerve	0.172	0.391	−0.102	0.612	−0.024	0.904
	Ulnar nerve	0.126	0.531	−0.218	0.274	0.120	0.552
	Tibial nerve	0.108	0.608	0.214	0.304	−0.515	0.008
	Peroneal nerve	−0.029	0.894	0.220	0.301	−0.541	0.006
SNAP amp. (μV)	Median nerve	0.099	0.622	−0.096	0.632	−0.184	0.359
	Ulnar nerve	0.097	0.630	−0.257	0.195	−0.199	0.319
	Sural nerve	−0.259	0.192	0.133	0.509	−0.655	<0.001
SCV (m/s)	Median nerve	0.336	0.137	0.026	0.911	−0.059	0.801
	Ulnar nerve	0.147	0.525	−0.307	0.175	0.026	0.911
	Sural nerve	0.186	0.407	0.530	0.011	−0.494	0.019

Abbreviations: CMAP amp, compound muscle action potential amplitude; DL, distal latency; MCV, motor nerve conduction velocity; NfL, neurofilament light chain; SCV, sensory nerve conduction velocity; SNAP amp, sensory nerve action potential amplitude.

No significant correlations among the *N*‐glycans, *O*‐glycans, and serum NfL levels were observed (*N*‐ and *O*‐glycans, *rs* = 0.295 [*p* = 0.135]; *N*‐glycans and serum NfL, *rs* = 0.153, [*p* = 0.447]; *O*‐glycans and serum NfL, *rs* = −0.294 [*p* = 0.137]).

## DISCUSSION

In the present study, we performed a comprehensive analysis of the serum glycans of treatment‐naïve patients with typical CIDP. We have discovered that the serum total *N*‐glycans, specifically the sialylated types, were significantly lower in the patients with typical CIDP than in the HC. Furthermore, the lower levels of total *N*‐glycans, particularly α2,6‐sialylated *N*‐glycans, and total *O*‐glycans in this category of patients demonstrated a reduced responsiveness to treatment over 12 weeks. This study reveals the potential of these glycans as novel biomarkers to determine the responsiveness to treatment.

In multiple sclerosis, *N*‐glycans play a crucial role in regulating oligodendrocytes and promoting myelin sheath formation and repair. [16, 23, 24, 25, 26, 27] In the serum of patients with progressive multiple sclerosis, a significant decrease in serum HexNAc, which is a stereoisomer of GlcNAc necessary for branching *N*‐glycans, was observed. Lower levels of serum HexNAc have been reported to be positively correlated with disease severity [16]. *N*‐glycans were proven to suppress the responses of inflammatory type 1 and type 17 helper T cells as well as activity of inflammatory B‐cells in an inflammatory demyelination mouse model [25]. Moreover, the oral supplementation of GlcNAc promoted remyelination and has been reported to exhibit neuroprotective effects for demyelinated axons in a mouse model [26]. These studies suggest that a decrease in total serum *N*‐glycan levels can serve as a potential biomarker of inflammation in the peripheral nervous system as well.

Regarding CIDP, the decreased levels of sialylated *N*‐glycans within the IgG‐Fc portion of the CIDP patients' serum have been observed, revealing its association with disease severity [10]. Furthermore, the presence of sialic acid in the *N*‐glycans of IgG‐Fc has been confirmed to compromise the effector function of inflammatory IgG, specifically by inhibiting complement‐mediated cytotoxicity [9]. The present study provides novel insights into the role of glycans in the development of CIDP: the decrease in sialylated *N*‐glycans is indicative of an inflammatory pathophysiology in CIDP, affecting not only IgG‐Fc but also the entire structure of glycoproteins.

Research on the association between therapeutic responsiveness to IVIg and serum glycosylation is limited. In the previous study, when IVIg was administered to the patients with CIDP, a significant elevation in serum sialylated IgG‐Fc was observed showing a clinical improvement of the condition [10]. Moreover, α2,6‐linked sialylated *N*‐glycans have been reported to be expressed more in M2 macrophages than in M1 macrophages and exhibit anti‐inflammatory effects [28]. In the present study, we discovered that the levels of α2,6‐linked sialylated glycans were strongly associated with therapeutic efficacy of the 12‐week treatment, including IVIg.

In contrast to *N*‐glycans, no significant difference was observed in the levels of *O*‐glycans between the patients with typical CIDP and HC. Nevertheless, lower levels of *O*‐glycans were linked with resistance to therapy over 12 weeks. The dysregulation in the biosynthesis of *O*‐glycans has been reported to play a crucial role in inflammation, infection, and carcinogenesis [29, 30]. Mice deficient in O‐GlcNAcylation of Schwann cells did not form normal myelin and develop demyelinating peripheral neuropathy [31]. However, only a few studies have been conducted on the role of *O*‐glycans in peripheral nerve disorders, and several aspects remain unclear. Thus, further research in this area is required.

Serum NfL has been reported to be a useful biomarker reflecting axonal damage in CIDP [18, 32]. In the present study, the serum NfL levels were discovered to be negatively correlated with CMAP amplitude in the nerves within the upper and lower limbs, possibly revealing axonal degeneration. An increase in serum NfL was confirmed to be associated with disease progression 1 year after serum sampling in patients with CIDP but has not been shown to reflect short‐term treatment responses after IVIg administration [33, 34, 35]. In our study, we also confirmed that serum NfL was not associated with the treatment responsiveness over 12 weeks. Instead, the levels of serum glycans were associated with short‐term response to treatment.

However, this study has several limitations. First, because of its exploratory nature and limited sample size, our findings should be interpreted with caution. The validation of our clinical observations can be strengthened by conducting further research with a larger cohort. Moreover, the mRS, which is assessed in all patients before and after treatment, was applied to assess severity, but other clinical outcomes such as the Inflammatory Neuropathy Cause and Treatment disability score, Inflammatory Rasch‐Built Overall Disability scale score, and grip strength were not assessed. Furthermore, as this is a retrospective cohort study and post‐treatment evaluations of serum glycans were missing, long‐term changes in glycan profiles remain uncertain. A comparison of glycan levels before and after treatment could provide valuable insights into the relationship between glycan changes and the magnitude of the treatment response. Finally, as the study focused exclusively on Japanese patients, glycans need to be measured in other ethnic groups to validate this marker.

In conclusion, our study identified low levels of serum total *N*‐glycans, specifically the sialylated types, as potential glycan biomarkers for typical CIDP. These findings are closely related to the pathogenesis of CIDP. Further studies are needed to develop a more convenient assay system for measuring changes in serum glycans in CIDP and to validate these findings in a larger sample size.

## AUTHOR CONTRIBUTIONS

**Soma Furukawa:** Conceptualization; writing – review and editing; writing – original draft; visualization; methodology; software; investigation; validation; formal analysis; project administration; data curation; resources. **Yuki Fukami:** Conceptualization; methodology; software; investigation; validation; formal analysis; supervision; project administration; visualization; funding acquisition; writing – original draft; writing – review and editing. **Hisatoshi Hanamatsu:** Conceptualization; methodology; visualization; writing – original draft; writing – review and editing; data curation; formal analysis; resources; validation. **Ikuko Yokota:** Methodology; data curation; resources. **Jun‐ichi Furukawa:** Conceptualization; methodology; writing – original draft; visualization; writing – review and editing; funding acquisition; formal analysis; data curation; resources; validation. **Masaya Hane:** Data curation; resources; methodology. **Ken Kitajima:** Methodology; data curation; resources. **Chihiro Sato:** Methodology; supervision; data curation; resources; conceptualization. **Keita Hiraga:** Resources; conceptualization. **Yuki Satake:** Resources. **Satoru Yagi:** Conceptualization; resources. **Haruki Koike:** Conceptualization; methodology; supervision; writing – original draft; investigation; data curation. **Masahisa Katsuno:** Conceptualization; methodology; software; data curation; investigation; validation; formal analysis; supervision; project administration; visualization; funding acquisition; writing – original draft; writing – review and editing; resources.

## FUNDING INFORMATION

This work was supported in part by JSPS KAKENHI Grant Numbers JP23K14751 (Y.F.), JP22H03502 (J.F.), and JP23H00420 (M.K.). Part of this study was conducted under the Human Glycome Atlas Project (HGA).

## CONFLICT OF INTEREST STATEMENT

Drs. S. Furukawa, H. Hanamatsu, I. Yokota, M. Hane, K. Kitajima, C. Sato, K. Hiraga, Y. Satake, S. Yagi and H. Koike report no disclosures. Dr. J. Furukawa is supported by a JSPS KAKENHI Grant Number JP22H03502. Dr. Y. Fukami is supported by a JSPS KAKENHI Grant Number JP23K14751. Dr. M. Katsuno is supported by a JSPS KAKENHI Grant Number JP23H00420 and AMED under Grant Numbers JP21wm0425013, JP23ek0109652, and 24lk0221191.

## Supporting information


**Appendix S1:** Supporting Information.

## Data Availability

Anonymized data not published within this article will be made available upon request from any qualified investigator.
