# Supplementary material for: Serum glycobiomarkers for chronic inflammatory demyelinating polyneuropathy
Source: Eur J Neurol. 2024 Dec 25;32(1):e70023. doi: 10.1111/ene.70023 (PMC11669748; doi:10.1111/ene.70023)
Supplement: Supplementary file 1 — Appendix S1: Supporting Information. [file ENE-32-e70023-s001.docx]

**Supplemental Data**

**Serum Glycobiomarkers for Chronic Inflammatory Demyelinating Polyneuropathy**

Soma Furukawa, Yuki Fukami, Hisatoshi Hanamatsu, Ikuko Yokota, Jun-ichi Furukawa, Masaya Hane, Ken Kitajima, Chihiro Sato, Keita Hiraga, Yuki Satake, Satoru Yagi, Haruki Koike, Masahisa Katsuno

**Contents:**

Two Supplemental Tables (Tables S1, Tables S2)

Six Supplemental Figure (Figure S1, Figure S2, Figure S3, Figure S4, Figure S5, Figure S6)

***Corresponding author**: Masahisa Katsuno or Yuki Fukami

Masahisa Katsuno, MD, PhD

Department of Neurology, Nagoya University Graduate School of Medicine,65　Tsurumai-Cho, Showa-ku, Nagoya, Aichi, 466-8550, Japan. Tel: +81-52-744-2389; Fax: +81-52-744-2384, e-mail: [katsuno.masahisa.i1@f.mail.nagoya-u.ac.jp](mailto:katsuno.masahisa.i1@f.mail.nagoya-u.ac.jp)

Yuki Fukami, MD, PhD

Department of Neurology, Nagoya University Graduate School of Medicine,65　Tsurumai-Cho, Showa-ku, Nagoya, Aichi, 466-8550, Japan. Tel: +81-52-744-2391; Fax: +81-52-744-2393, e-mail: fukami.yuki.t2@f.mail.nagoya-u.ac.jp

**Figure S1**: Flow diagram of the study subject enrollment


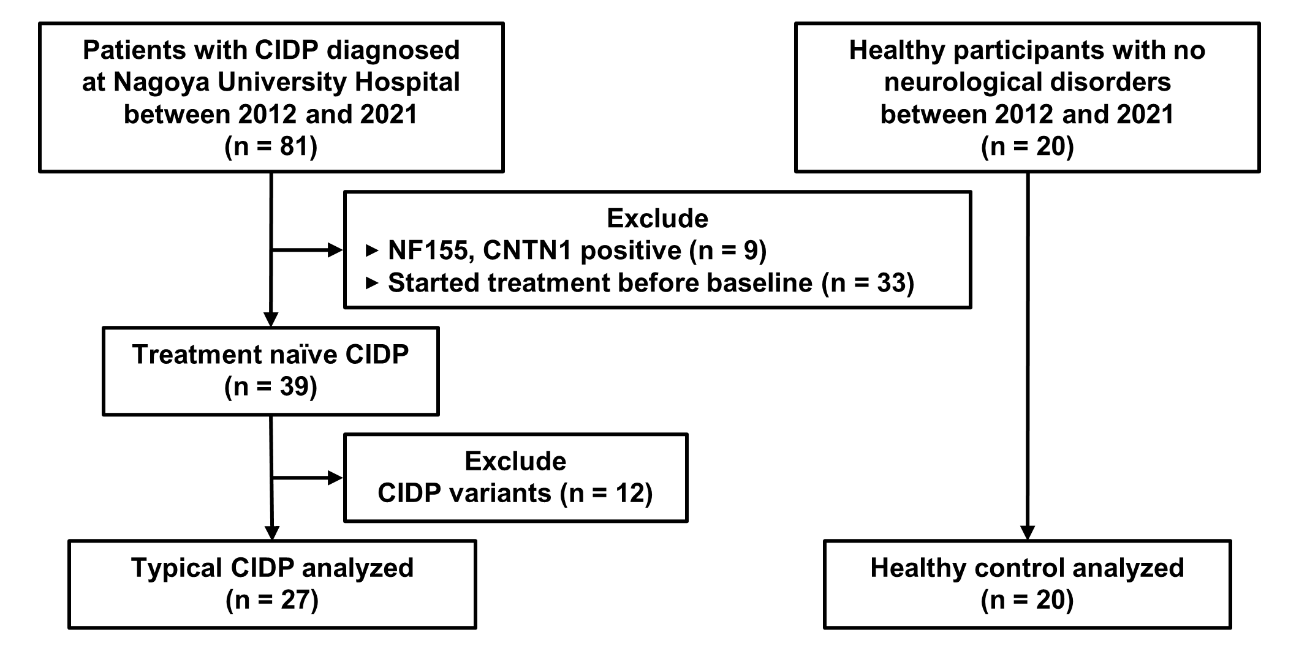


Enrollment flow diagram for patients with CIDP and HC. CIDP, chronic inflammatory demyelinating polyneuropathy. NF155, anti-neurofascin155 antibody; CNTN1, anti-contactin1 antibody.

**Tables S1**: *N*-glycan Profile in serum of healthy controls and CIDP patients

| No. | m/z | Chemical composition | HC Mean  ± SD  pmol/ μL | CIDP Mean  ± SD  pmol/ μL | *p* value |
| --- | --- | --- | --- | --- | --- |
| **high mannose *N*-glycans** | | | | | |
| 1 | 1664.6536 | (Hex)2 + (Man)3(GlcNAc)2 | 0.3525 ± 0.2953 | 0.4368 ± 0.4813 | 0.763 |
| 2 | 1826.7064 | (Hex)3 + (Man)3(GlcNAc)2 | 0.5634 ± 0.3455 | 0.4948 ± 0.3810 | 0.606 |
| 3 | 1988.7592 | (Hex)4 + (Man)3(GlcNAc)2 | 0.082 ± 0.0825 | 0.045 ± 0.0599 | 0.111 |
| 4 | 2150.812 | (Hex)5 + (Man)3(GlcNAc)2 | 0.1167 ± 0.1181 | 0.0987 ± 0.1386 | 0.411 |
| 5 | 2312.8648 | (Hex)6 + (Man)3(GlcNAc)2 | 0.5538 ± 0.2200 | 0.3851 ± 0.2454 | <0.05 |
| **neutral *N*-glycans** | | | | | |
| 6 | 1746.7068 | (HexNAc)2 + (Man)3(GlcNAc)2 | 0.0122 ± 0.0312 | 0.0047 ± 0.0171 | 0.387 |
| 7 | 1867.733 | (Hex)2 (HexNAc)1 + (Man)3(GlcNAc)2 | 0.0051 ± 0.0229 | 0.0027 ± 0.0142 | 0.806 |
| 8 | 1892.7647 | (HexNAc)2 (Fuc)1 + (Man)3(GlcNAc)2 | 13.3015 ± 15.4073 | 22.6832 ± 21.9390 | <0.05 |
| 9 | 1908.7596 | (Hex)1 (HexNAc)2 + (Man)3(GlcNAc)2 | 0.1554 ± 0.1818 | 0.1030 ± 0.1223 | 0.464 |
| 10 | 1949.7862 | (HexNAc)3 + (Man)3(GlcNAc)2 | 0.0338 ± 0.122 | 0.0392 ± 0.0811 | 0.321 |
| 11 | 2029.7858 | (Hex)3 (HexNAc)1 + (Man)3(GlcNAc)2 | 0.0168 ± 0.0421 | 0.0094 ± 0.0276 | 0.603 |
| 12 | 2054.8175 | (Hex)1 (HexNAc)2 (Fuc)1 + (Man)3(GlcNAc)2 | 30.6019 ± 15.9878 | 36.5518 ± 18.9787 | 0.322 |
| 13 | 2070.8124 | (Hex)2 (HexNAc)2 + (Man)3(GlcNAc)2 | 0.5108 ± 0.2852 | 0.3511 ± 0.2185 | 0.121 |
| 14 | 2095.8441 | (HexNAc)3 (Fuc)1 + (Man)3(GlcNAc)2 | 1.4634 ± 1.714 | 2.0658 ± 2.2047 | 0.138 |
| 15 | 2111.839 | (Hex)1 (HexNAc)3 + (Man)3(GlcNAc)2 | 0.0334 ± 0.0721 | 0.0802 ± 0.1574 | 0.313 |
| 16 | 2216.8703 | (Hex)2 (HexNAc)2 (Fuc)1 + (Man)3(GlcNAc)2 | 14.9131 ± 8.593 | 12.8980 ± 7.1733 | 0.478 |
| 17 | 2257.8969 | (Hex)1 (HexNAc)3 (Fuc)1 + (Man)3(GlcNAc)2 | 3.7824 ± 2.9545 | 4.4245 ± 3.2181 | 0.282 |
| 18 | 2273.8918 | (Hex)2(HexNAc)3+(Man)3(GlcNAc)2 | 0.0227 ± 0.0567 | 0.0113 ± 0.0413 | 0.410 |
| 19 | 2419.9497 | (Hex)2 (HexNAc)3 (Fuc)1 + (Man)3(GlcNAc)2 | 2.0514 ± 1.1238 | 1.4388 ± 0.7359 | <0.05 |
| 20 | 2582.0025 | (Hex)3 (HexNAc)3 (Fuc)1 + (Man)3(GlcNAc)2 | 0.0087 ± 0.0389 | 0.0000 ± 0.0000 | 0.245 |
| **sialylated *N*-glycans** | | | | | |
| 21 | 2374.94366 | (Hex)2 (HexNAc)2 (NeuAc)1[a2,3] + (Man)3(GlcNAc)2 | 0.8252 ± 0.3683 | 0.6237 ± 0.2533 | <0.05 |
| 22 | 2505.00666 | (Hex)1 (HexNAc)2 (Fuc)2 (NeuAc)1[a2,3] + (Man)3(GlcNAc)2 | 0.0000 ± 0.0000 | 0.0169 ± 0.0441 | 0.075 |
| 23 | 2521.00156 | (Hex)2 (HexNAc)2 (Fuc)1 (NeuAc)1[a2,3] + (Man)3(GlcNAc)2 | 1.0061 ± 0.2782 | 0.7570 ± 0.2099 | <0.01 |
| 24 | 2667.05946 | (Hex)2 (HexNAc)2 (Fuc)2 (NeuAc)1[a2,3] + (Man)3(GlcNAc)2 | 0.0000 ± 0.0000 | 0.0057 ± 0.0295 | 0.389 |
| 25 | 2679.07492 | (Hex)2 (HexNAc)2 (NeuAc)2[a2,3/a2,3] + (Man)3(GlcNAc)2 | 0.3476 ± 0.0853 | 0.1694 ± 0.1619 | <0.001 |
| 26 | 2724.08096 | (Hex)2 (HexNAc)3 (Fuc)1 (NeuAc)1[a2,3] + (Man)3(GlcNAc)2 | 0.0505 ± 0.0914 | 0.0120 ± 0.0439 | 0.086 |
| 27 | 2825.13282 | (Hex)2 (HexNAc)2 (Fuc)1 (NeuAc)2[a2,3/a2,3] + (Man)3(GlcNAc)2 | 0.7992 ± 0.1877 | 0.5869 ± 0.1915 | <0.001 |
| 28 | 1875.78996 | (HexNAc)1 (NeuAc)1[a2,6] + (Man)3(GlcNAc)2 | 0.0159 ± 0.0406 | 0.0038 ± 0.0145 | 0.345 |
| 29 | 2037.84276 | (Hex)1 (HexNAc)1 (NeuAc)1[a2,6] + (Man)3(GlcNAc)2 | 0.2807 ± 0.1829 | 0.1983 ± 0.1652 | 0.116 |
| 30 | 2183.90066 | (Hex)1 (HexNAc)1 (Fuc)1 (NeuAc)1[a2,6]+ (Man)3(GlcNAc)2 | 0.0046 ± 0.0206 | 0.0078 ± 0.0282 | 0.703 |
| 31 | 2199.89556 | (Hex)2 (HexNAc)1 (NeuAc)1[a2,6] + (Man)3(GlcNAc)2 | 0.5458 ± 0.2668 | 0.5266 ± 0.3044 | 0.667 |
| 32 | 2240.92216 | (Hex)1 (HexNAc)2 (NeuAc)1[a2,6] + (Man)3(GlcNAc)2 | 0.7768 ± 0.6772 | 0.7594 ± 0.6438 | 0.897 |
| 33 | 2361.94836 | (Hex)3 (HexNAc)1 (NeuAc)1[a2,6] + (Man)3(GlcNAc)2 | 0.3527 ± 0.1635 | 0.2463 ± 0.1817 | <0.05 |
| 34 | 2386.98006 | (Hex)1 (HexNAc)2 (Fuc)1 (NeuAc)1[a2,6] + (Man)3(GlcNAc)2 | 1.0207 ± 0.4618 | 1.1189 ± 0.6418 | 0.897 |
| 35 | 2402.97496 | (Hex)2 (HexNAc)2 (NeuAc)1[a2,6] + (Man)3(GlcNAc)2 | 94.7950 ± 39.4059 | 78.7563 ± 24.9703 | 0.175 |
| 36 | 2549.03286 | (Hex)2 (HexNAc)2 (Fuc)1 (NeuAc)1[a2,6] + (Man)3(GlcNAc)2 | 39.5540 ± 15.6253 | 32.5009 ± 12.3687 | 0.175 |
| 37 | 2565.02776 | (Hex)3 (HexNAc)2 (NeuAc)1[a2,6] + (Man)3(GlcNAc)2 | 0.3440 ± 0.3028 | 0.1603 ± 0.2165 | <0.05 |
| 38 | 2590.05946 | (Hex)1 (HexNAc)3 (Fuc)1 (NeuAc)1[a2,6] + (Man)3(GlcNAc)2 | 0.3705 ± 0.3625 | 0.5628 ± 0.7233 | 0.522 |
| 39 | 2606.05436 | (Hex)2 (HexNAc)3 (NeuAc)1[a2,6] + (Man)3(GlcNAc)2 | 1.3613 ± 1.1682 | 1.2988 ± 1.3874 | 0.505 |
| 40 | 2695.09076 | (Hex)2 (HexNAc)2 (Fuc)2 (NeuAc)1[a2,6] + (Man)3(GlcNAc)2 | 0.0000 ± 0.0000 | 0.07170 ± 0.3726 | 0.389 |
| 41 | 2735.13752 | (Hex)2 (HexNAc)2 (NeuAc)2[a2,6/a2,6] + (Man)3(GlcNAc)2 | 772.6195 ± 157.6319 | 662.1118 ± 165.3016 | <0.05 |
| 42 | 2752.11226 | (Hex)2 (HexNAc)3 (Fuc)1 (NeuAc)1[a2,6] + (Man)3(GlcNAc)2 | 23.7021 ± 14.1587 | 17.0229 ± 9.9946 | 0.074 |
| 43 | 2768.10716 | (Hex)3 (HexNAc)3 (NeuAc)1[a2,6] + (Man)3(GlcNAc)2 | 1.9139 ± 0.9426 | 1.0670 ± 0.8065 | <0.01 |
| 44 | 2881.19542 | (Hex)2 (HexNAc)2 (Fuc)1 (NeuAc)2[a2,6/a2,6] + (Man)3(GlcNAc)2 | 20.9835 ± 10.0858 | 19.4358 ± 9.1872 | 0.505 |
| 45 | 2914.16506 | (Hex)3 (HexNAc)3 (Fuc)1 (NeuAc)1[a2,6] + (Man)3(GlcNAc)2 | 0.1600 ± 0.1184 | 0.0888 ± 0.0890 | <0.05 |
| 46 | 2938.21692 | (Hex)2 (HexNAc)3 (NeuAc)2[a2,6] + (Man)3(GlcNAc)2 | 0.0634 ± 0.1044 | 0.0249 ± 0.0616 | 0.167 |
| 47 | 3084.27482 | (Hex)2 (HexNAc)3 (Fuc)1 (NeuAc)2[a2,6/a2,6] + (Man)3(GlcNAc)2 | 7.8254 ± 7.1791 | 9.684 ± 12.4826 | 0.830 |
| 48 | 3100.26972 | (Hex)3 (HexNAc)3 (NeuAc)2[a2,6/a2,6] + (Man)3(GlcNAc)2 | 0.8090 ± 0.4930 | 0.5330 ± 0.4242 | <0.05 |
| 49 | 3246.32762 | (Hex)3 (HexNAc)3 (Fuc)1 (NeuAc)2[a2,6/a2,6] + (Man)3(GlcNAc)2 | 0.0312 ± 0.1063 | 0.0138 ± 0.0500 | 0.722 |
| 50 | 3432.43228 | (Hex)3 (HexNAc)3 (NeuAc)3[a2,6/a2,6/a2,6] + (Man)3(GlcNAc)2 | 3.0262 ± 2.5515 | 1.7734 ± 1.4863 | <0.05 |
| 51 | 2707.10622 | (Hex)2 (HexNAc)2 (NeuAc)2[a2,3/a2,6] + (Man)3(GlcNAc)2 | 66.9269 ± 18.8389 | 55.8431 ± 22.2790 | <0.05 |
| 52 | 2853.16412 | (Hex)2 (HexNAc)2 (Fuc)1 (NeuAc)2[a2,3/a2,6] + (Man)3(GlcNAc)2 | 2.1842 ± 1.0207 | 2.0977 ± 1.0568 | 0.846 |
| 53 | 3056.24352 | (Hex)2 (HexNAc)3 (Fuc)1 (NeuAc)2[a2,3/a2,6] + (Man)3(GlcNAc)2 | 0.0611 ± 0.1119 | 0.0358 ± 0.1150 | 0.237 |
| 54 | 3072.23842 | (Hex)3 (HexNAc)3 (NeuAc)2[a2,3/a2,6] + (Man)3(GlcNAc)2 | 1.2570 ± 0.7070 | 0.9688 ± 0.7841 | 0.085 |
| 55 | 3218.29632 | (Hex)3 (HexNAc)3 (Fuc)1 (NeuAc)2[a2,3/a2,6] + (Man)3(GlcNAc)2 | 0.0810 ± 0.1690 | 0.0464 ± 0.0887 | 0.662 |
| 56 | 3376.36968 | (Hex)3 (HexNAc)3 (NeuAc)3[a2,3/a2,3/a2,6] + (Man)3(GlcNAc)2 | 0.8991 ± 0.5469 | 0.6795 ± 0.6542 | 0.085 |
| 57 | 3404.40098 | (Hex)3 (HexNAc)3 (NeuAc)3[a2,3/a2,6/a2,6] + (Man)3(GlcNAc)2 | 34.7527 ± 19.0509 | 23.2955 ± 17.2103 | 0.058 |
| 58 | 3522.42758 | (Hex)3 (HexNAc)3 (Fuc)1 (NeuAc)3[a2,3/a2,3/a2,6] + (Man)3(GlcNAc)2 | 0.1016 ± 0.1820 | 0.0809 ± 0.1175 | 0.867 |
| 59 | 3550.45888 | (Hex)3 (HexNAc)3 (Fuc)1 (NeuAc)3[a2,3/a2,6/a2,6] + (Man)3(GlcNAc)2 | 6.4372 ± 9.9546 | 6.2858 ± 6.7304 | 0.846 |

P-values were calculated using the Mann-Whitney U test between the HC and CIDP groups (significance level α = 0.05). CIDP chronic inflammatory demyelinating polyradiculoneuropathy; HC, healty controls; m/z, mass-to-charge ratio.

**Tables S2**: *O*-glycan Profile in serum of healthy controls and CIDP patients

| No. | m/z | Chemical composition | HC Mean  ± SD pmol/ μL | CIDP Mean  ± SD pmol/ μL | *p* value |
| --- | --- | --- | --- | --- | --- |
| **neutral *O*-glycans** | | | | | |
| 1 | 503.1909 | Xly | 0.0854 ± 0.0391 | 0.0778 ± 0.0436 | 0.547 |
| 2 | 533.2012 | Hex | 0.0278 ± 0.0446 | 0.0422 ± 0.0651 | 0.591 |
| 3 | 574.2280 | HexNAc | 0.0369 ± 0.0669 | 0.0507 ± 0.0757 | 0.595 |
| 4 | 736.2806 | (Hex)1(HexNAc)1 | 0.7747 ± 0.2619 | 0.9759 ± 0.4052 | 0.111 |
| 5 | 939.3600 | (Hex)1(HexNAc)2 | 0.0000 ± 0.0000 | 0.0000 ± 0.0000 | 1.000 |
| 6 | 1101.4126 | (Hex)2(HexNAc)2 | 0.0493 ± 0.0430 | 0.0400 ± 0.0442 | 0.509 |
| 7 | 1247.4705 | (Hex)2(HexNAc)2(Fuc)1 | 0.0279 ± 0.0333 | 0.0106 ± 0.0237 | <0.05 |
| **sialylated *O*-glycans** | | | | | |
| 8 | 906.3863 | (HexNAc)1(6NeuAc)1[a2,6] | 0.0161 ± 0.0309 | 0.0167 ± 0.0413 | 0.562 |
| 9 | 1040.4076 | (HexNAc)1(Hex)1(3NeuAc)1[a2,3] | 26.3276 ± 5.9795 | 23.4743 ± 7.4477 | 0.182 |
| 10 | 1056.4025 | (HexNAc)1(Hex)1(3NeuGc)1[a2,3] | 0.1930 ± 0.0542 | 0.2121 ± 0.0901 | 0.333 |
| 11 | 1068.4389 | (HexNAc)1(Hex)1(6NeuAc)1[a2,6] | 0.3883 ± 0.0694 | 0.4433 ± 0.1240 | 0.102 |
| 12 | 1372.5659 | (HexNAc)1(Hex)1(6NeuAc)1(3NeuAc)1　[a2,3/a2,6] | 5.0641 ± 1.2535 | 4.9766 ± 1.6104 | 0.813 |
| 13 | 1388.5608 | (HexNAc)1(Hex)1(6NeuAc)1(3NeuGc)1　[a2,3/a2,6] | 0.0366 ± 0.0446 | 0.0270 ± 0.0446 | 0.345 |
| 14 | 1243.4870 | (Hex)1(HexNAc)2(3NeuAc)1[a2,3] | 0.0398 ± 0.0329 | 0.0246 ± 0.0391 | 0.107 |
| 15 | 1405.5396 | (HexNAc)2(Hex)2(3NeuAc)1[a2,3] | 0.1836 ± 0.0447 | 0.2517 ± 0.0965 | <0.01 |
| 16 | 1551.5975 | (HexNAc)2(Hex)2(3NeuAc)1(Fuc)1[a2,3] | 0.0288 ± 0.0294 | 0.0135 ± 0.0271 | <0.05 |
| 17 | 1709.6666 | (HexNAc)2(Hex)2(3NeuAc)2[a2,3] | 0.4163 ± 0.1077 | 0.5088 ± 0.1669 | <0.05 |
| 18 | 1855.7245 | (HexNAc)2(Hex)2(3NeuAc)2(Fuc)1[a2,3] | 0.0277 ± 0.0390 | 0.0290 ± 0.0508 | 0.726 |

P-values were calculated using the Mann-Whitney U test between the HC and CIDP groups (significance level α = 0.05). m/z, mass-to-charge ratio.

**Figure S2**: Association of serum *N*-glycans levels with response after 2 weeks of initial treatment

**A.** Association between the serum *N*-glycans levels and mRS improvement score from baseline after 2 weeks of initial treatment in patients with CIDP. The significant difference in serum total *N*-glycans levels was observed among the three groups with mRS improvement scores of 0, 1, and 2 (801.1 [720.0–969.8], 1032.9 [878.9–1114.0], and 1163.2 [967.7–1405.4], respectively, *p* < 0.05). **B.** ROC analysis differentiating the responder group from the non-responder group showed that the AUC for the serum total *N*-glycans and sialylated *N*-glycans levels were 0.827 (95% CI, 0.622–1.000, *p* < 0.01) and 0.840 (95% CI, 0.640–1.000, *p* < 0.01), respectively. The top and bottom edges of each box indicate the IQR. The I-bar indicates the range between the minimum and maximum values. **p* < 0.05, multiple comparisons using Bonferroni correction. ΔmRS, degree of mRS improvement after 2 weeks of initial treatment.

**Figure S3**: Association of serum *O*-glycans levels with response after 2 weeks of initial treatment

**A**. Association between the serum *O*-glycans levels and mRS improvement score from baseline after 2 weeks of initial treatment in patients with CIDP. There was a significant difference in serum total *O*-glycans levels among the three groups with mRS improvement scores of 0, 1, and 2 (23.8 [15.1–32.6], 30.1 [27.3–39.0], and 41.2 [36.7–44.6], respectively, *p* < 0.01). **B.** ROC analysis to discriminate the responder group from the non-responder group showed that the AUC for the serum total *O*-glycans and sialylated *O*-glycans levels were 0.846 (95% CI, 0.684–1.000, *p* < 0.01) and 0.846 (95% CI, 0.684–1.000, *p* < 0.01), respectively. The top and bottom edges of each box indicate the IQR. The I-bar indicates the range between the minimum and maximum values. ***p* < 0.01, multiple comparisons using Bonferroni correction. ΔmRS, degree of mRS improvement after 2 weeks of initial treatment.

**Figure S4**: Association of serum *N*-glycans levels with response after 4 weeks of initial treatment


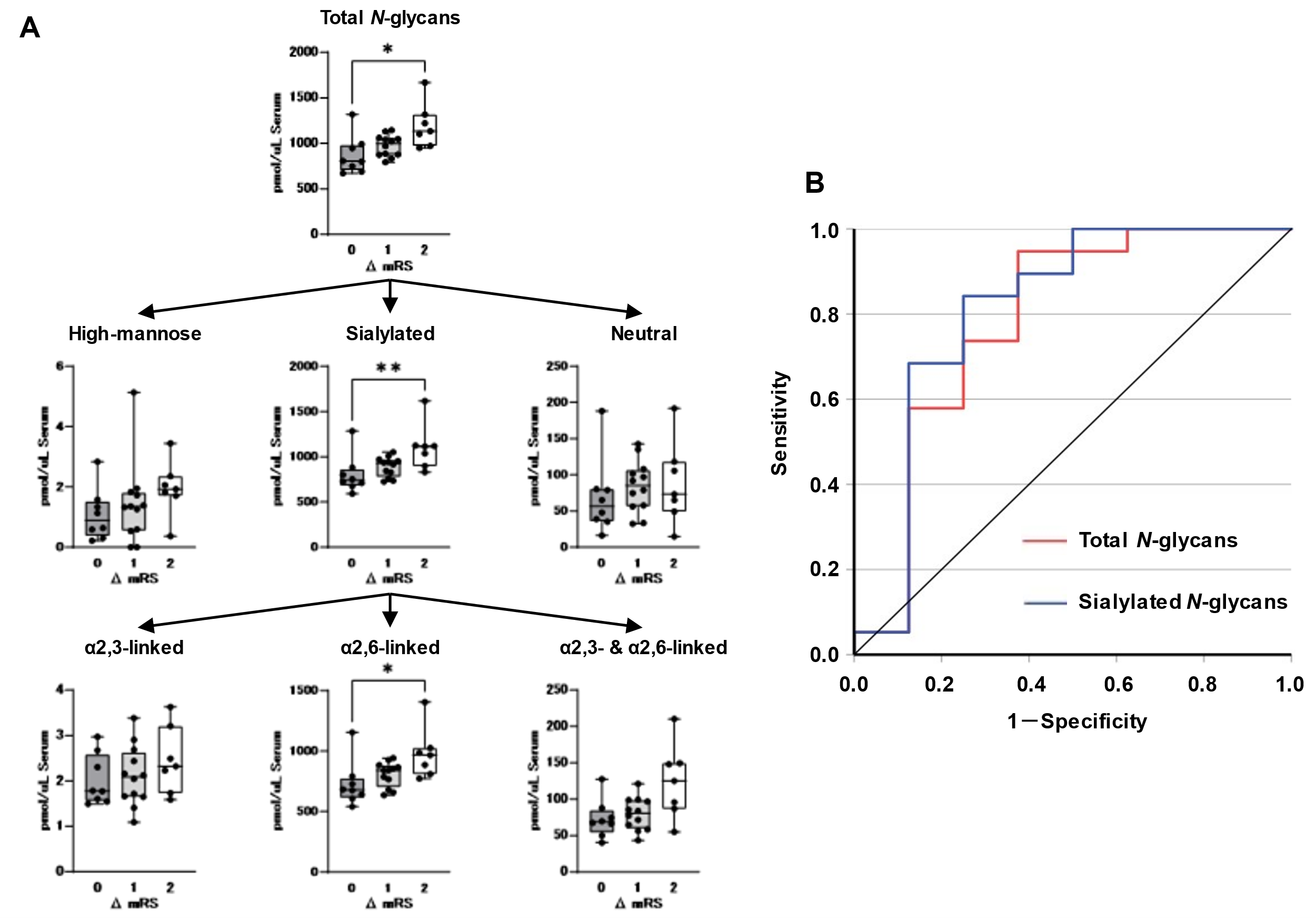


**A.** Association between the serum *N*-glycans levels and mRS improvement score from baseline after 4 weeks of initial treatment in patients with CIDP. The significant difference in serum total *N*-glycans levels was observed among the three groups with mRS improvement scores of 0, 1, and 2 (804.0 [705.3–980.9], 997.9 [874.6–1058.9], and 1133.5 [973.3–1317.5], respectively, *p* < 0.05). **B.** ROC analysis differentiating the responder group from the non-responder group showed that the AUC for the serum total *N*-glycans and sialylated *N*-glycans levels were 0.783 (95% CI, 0.555–1.000, *p* < 0.05) and 0.809 (95% CI, 0.588–1.000, *p* < 0.05), respectively. The top and bottom edges of each box indicate the IQR. The I-bar indicates the range between the minimum and maximum values. **p* < 0.05, ***p* < 0.01, multiple comparisons using Bonferroni correction. ΔmRS, degree of mRS improvement after 4 weeks of initial treatment.

**Figure S5**: Association of serum *O*-glycans levels with response after 4 weeks of initial treatment


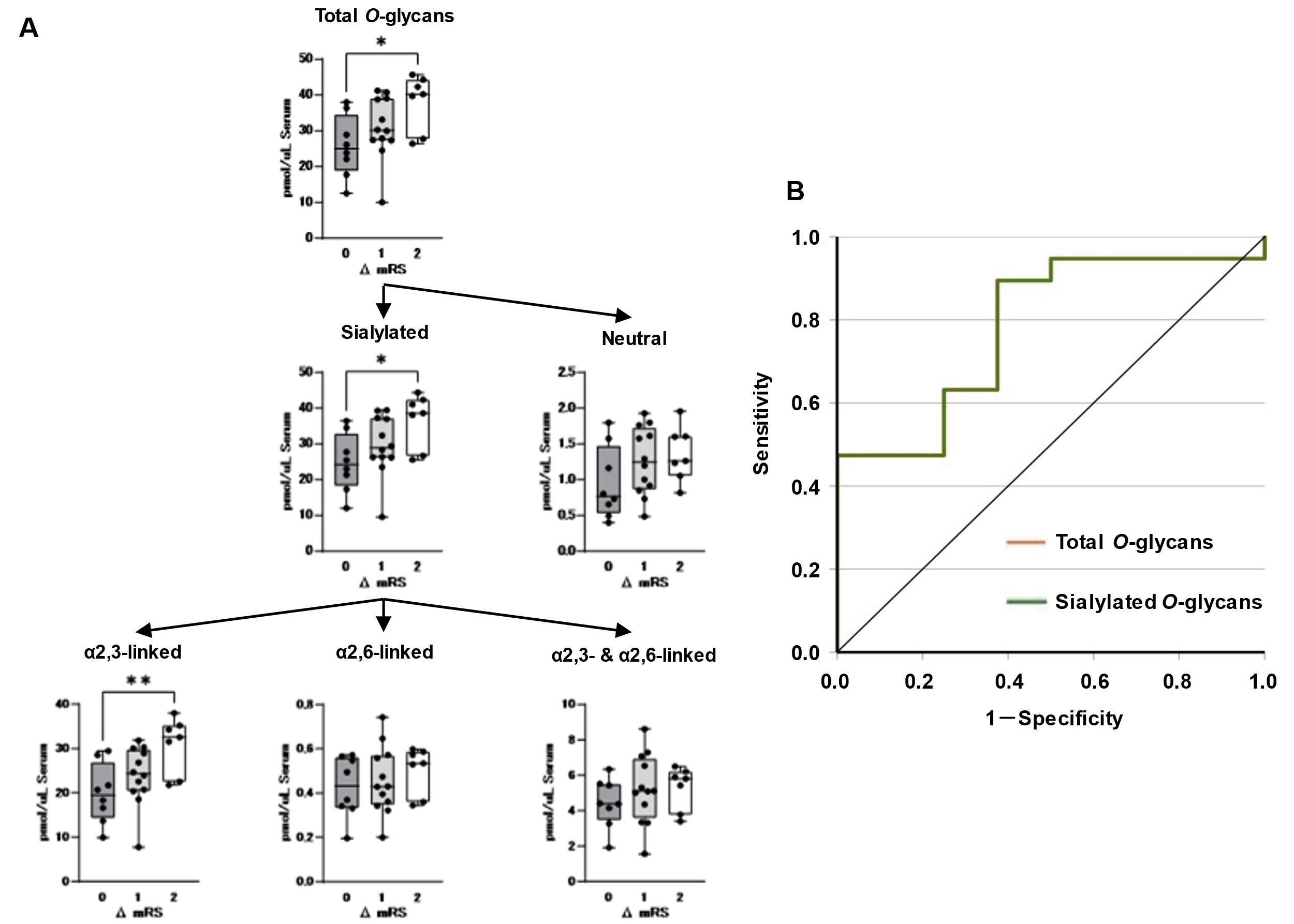


**A**. Association between the serum *O*-glycans levels and mRS improvement score from baseline after 4 weeks of initial treatment in patients with CIDP. There was a significant difference in serum total *O*-glycans levels among the three groups with mRS improvement scores of 0, 1, and 2 (25.0 [18.8–34.5], 30.1 [27.3–39.0], and 40.2 [27.7–44.3], respectively, *p* < 0.05). **B.** ROC analysis to discriminate the responder group from the non-responder group showed that the AUC for the serum total *O*-glycans and sialylated *O*-glycans levels were 0.783 (95% CI, 0.596–0.970, *p* < 0.05) and 0.783 (95% CI, 0.596–0.970, *p* < 0.05), respectively. The top and bottom edges of each box indicate the IQR. The I-bar indicates the range between the minimum and maximum values. **p* < 0.05, ***p* < 0.01, multiple comparisons using Bonferroni correction. ΔmRS, degree of mRS improvement after 4 weeks of initial treatment.

**Figure S6**: Association of factors other than glycans with the initial response to treatment with IVIg.

B

A

C

D

Δ mRS

H

E

F

G

**A­­­­–H.** There were no significant differences in age (A), duration of disease (B), CMAP amplitudes of each nerve (C–F), CSF protein (G), nor serum NfL levels (H) among the three groups with mRS improvements of 0, 1 and 2. Statistical analysis was performed using the Kruskal–Wallis test. Horizontal lines in the boxplots indicate the median. The top and bottom edges of each box indicate the IQR. The I-bar indicates the range between the minimum and maximum values. ΔmRS, degree of mRS improvement after 2 weeks of initial IVIg. No statistically significant difference was shown in any comparison.
